# Case Report: High doses of Zolpidem and QT interval lengthening: Is there a relationship? A case series

**DOI:** 10.3389/fpsyt.2022.1033061

**Published:** 2022-10-20

**Authors:** Simone Campagnari, Lorenzo Zamboni, Francesca Fusina, Rebecca Casari, Fabio Lugoboni

**Affiliations:** ^1^Unit of Addiction Medicine, Department of Internal Medicine, G.B. Rossi Hospital, Verona, Italy; ^2^Department of Neuroscience, Biomedicine and Movement, University of Verona, Verona, Italy; ^3^Department of General Psychology, University of Padua, Padua, Italy; ^4^Padova Neuroscience Center, University of Padua, Padua, Italy

**Keywords:** Zolpidem, abuse, high dose, addiction, QTc, Z-drug

## Abstract

Zolpidem is indicated in cases of severe insomnia in adults and, as for BDZs, its assumption should be limited to short periods under close medical supervision. Since several drugs cause corrected QT interval (QTc) elongation, the authors investigated whether high daily doses of Zolpidem could cause QTc elongation. The study was conducted in the Addiction Medicine Unit of the G.B. Rossi University Hospital in Verona. The data were collected from hospitalizations carried out between January 2015 and February 2020 and refer to a total of 74 patients, 38 males and 36 females, who were treated for detoxification from high doses of Zolpidem with the “Verona Detox Approach With Flumazenil.” One patient out of 74 had QTc elongation (479 ms). The patient was male and took a daily dose of 50 mg of Zolpidem; he did not take concomitant therapies that could cause QTc lengthening. He had no electrolyte alterations, no contemporary or previous intake of barbiturates, heroin, cocaine, THC, alcohol, NMDA or nicotine which could cause an elongation of the QTc interval. The present study highlights the low risk of QTc elongation due to high dosages of Zolpidem; however, if, on one hand, we can affirm that Zolpidem is a safe drug, on the other, the widespread use of high dosages of this drug for prolonged periods of time is problematic and worrying.

## Introduction

Benzodiazepines (BDZs) and Z-drugs (Zolpidem, Zaleplon, Zopiclone) are among the most prescribed drugs in Western countries and, while there has been a downward trend in annual BDZ prescriptions, Z-drug prescriptions have instead increased exponentially. This is probably due to a widespread opinion among doctors that the latter don’t involve the problems associated with chronic use of BDZs, while, in fact, Z-Drug (ZD) use requires the same safeguards that apply to BDZs (1–4).

The use of ZDs is indicated in cases of severe insomnia in adults and, as for BDZs, it should be limited to short periods under close medical supervision. The pharmacokinetic differences between the molecules allow for some important considerations with respect to the selection criteria of these drugs. Zolpidem and Zopiclone, being eliminated more slowly, are more suitable for the treatment of insomnia with central awakenings, unlike Zaleplon, which has a rapid elimination and is thus best suited for the management of initial insomnia (5). The main contraindications to the use of Z-drugs are the following: drug hypersensitivity, myasthenia gravis, severe respiratory insufficiency, Obstructive Sleep Apnea Syndrome (OSAS) and advanced hepatic insufficiency. The use of ZDs is contraindicated in children.

ZDs, like BDZs, are characterized by high manageability, and starting from their introduction on the market it was initially thought that they could offer advantages in terms of efficacy and safety compared to classic BDZs. However, over time it was shown that even this class of drugs can lead to important side effects, such as cognitive impairment, worsening of psychomotor performance, and increasing the risk of falls and fractures, especially if taken at higher doses or in association with other psychoactive substances such as alcohol. (6–9).

Zolpidem and Zopiclone abuse are primarily described in patients with a history of drug addiction, alcoholism or psychiatric disorders and, minimally, also in patients not belonging to these categories. Their use should, therefore, reflect careful indications concerning posology (10, 11).

Zolpidem is an imidazopyrimidine and, although it is not structurally connected to BDZs, it has a similar mechanism of action: it enhances the inhibitory effect of GABA on nerve transmission, binding the related receptor with consequent increases in permeability to chlorine ions.

Although early reports highlighted a profile of low abuse risk for Zolpidem, in recent years an important increase in Zolpidem dependence has been detected; for this reason, Zolpidem was transferred to Schedule IV of the 1971 Convention (i.e., for drugs inducing dependence such as BDZs) in 2001 (12–14).

High-dose Zolpidem use (600-2000 mg/die) has been associated with psychostimulant effects, such as feelings of well-being, euphoria (“high”), energy, alertness, sociability, talkativeness, delusions and psychotic experiences, sleepwalking, falling asleep while driving, sleep-related eating disorders or engaging in other activities while not fully awake (15–17).

It is also interesting to note that, in a large number of hypnotic drug abusers, a selective preference for Zolpidem was reported by subjects that were positive at screening tests for adult Attention Deficit/Hyperactivity Disorder (18, 19).

Finally, intense craving, inability to stop use and withdrawal were associated with long-term high dose Zolpidem consumption. Through the analysis of adverse reaction data provided by the European Medicines Agency and after assessing the potential for abuse and dependence of ZDs, it has been shown that Zolpidem is more frequently involved in both abuse and withdrawal problems.

The QT interval begins with the beginning of the QRS complex and ends with the end of the T wave and has an inverse relationship with heart rate (HR). A rate-related (or corrected) QT interval (QTc) according to Bazett’s formula (20) can be calculated as:


$$Q\,T\,c=\frac{\mathrm{QT}\,\mathrm{interval}}{\mathrm{cardiac}\,\mathrm{cycle}\,\mathrm{in}\text{second}}$$

The upper limit of a normal QTc interval is 470 ms in males and 480 ms in females. As for the lower limits of the QTc, they have not been well established, but sometimes values between 330 and 360 ms are mentioned. (21).

Long QT syndrome (LQTS) is a myocardial repolarization disorder characterized by a prolonged QT interval on the electrocardiogram (ECG). The main symptoms in patients presenting LQTS include palpitations, syncope, seizures and sudden cardiac death. This syndrome can be congenital or acquired. The acquired form is related to drug therapy and the presence of hypokalemia and hypomagnesaemia may accentuate the risk of drug-induced LQTS development. One of the main risks of LQTS is that it generates polymorphic ventricular tachycardia, i.e., a ventricular rhythm greater than 100 bpm with frequent changes in the QRS axis, its morphology, or both (22, 23).

Torsades de pointes (TdP) is a form of polymorphic ventricular tachycardia which derives from either acquired or congenital QT interval prolongation, and manifests with a heart rate between 160 and 250 bpm. These variations take the form of a progressive, sinusoidal and cyclical evolution of the QRS axis; the peaks of the QRS complexes appear to “twist” around the isoelectric line of the recording. This condition tends to spontaneously regress; however, multiple episodes can occur in rapid succession and may degenerate into ventricular fibrillation and sudden cardiac death. Determining the absolute and comparative risk of many drugs associated with QT interval prolongation is difficult, as most of the available data comes from case reports or small series of observations. Furthermore, the incidence of QT prolongation without torsades de pointes (Tdp) is much higher than the incidence of TdP itself.

The pathophysiological mechanism underlying drug-induced TdP is the development of abnormal depolarizations of the cell membrane in the final part of the action potential, defined as early post-depolarization (EAD), or during diastolic repolarization, termed late post-depolarization (DAD).

Almost all drugs that cause LQTS cause blockage of the potassium channel, thereby inhibiting the rapid outward flow of potassium ions and, therefore, cellular repolarization (24, 25).

Furthermore, lower heart rates result in a smaller potassium output from the cell during repolarization, as there are fewer repolarization events; also, the reduction of extracellular potassium increases the degree of inhibition induced by the drug on the rapid potassium current, consequently increasing the QT interval. (25).

Most patients with drug-induced LQT have one or more risk factors for the condition.

In a review of the literature that included 249 patients with non-cardiac drug-associated QT prolongation, 97% had at least one risk factor and 71% had at least two. These included: female sex in 71%, history of heart disease in 41%, concomitant use of another QT prolonging drug in 39%, hypokalemia in 28%, a high dose of the drug in 19%, and a previous history of LQTS in 18%.

The most common risk factor for drug-induced LQTS is being female. In a review of the literature of 332 patients with cardiovascular drug-associated Torsades de pointes (Tdp), 70% were women. Compared to males, females have a longer QTc and a greater response to drugs that block the rapid potassium channel, favoring Tdp, possibly due to the effect of sex steroids on ion channel expression. Estrogen potentiates the prolongation of the QT interval induced by bradycardia and the development of arrhythmia. Conversely, androgens reduce the QT interval and make it less sensitive to drugs. (26–28).

In scientific literature, benzodiazepines and Z-Drugs are considered safe concerning LQTS (29); to date, however, there are no studies which address the abuse of high dosage of Z-drugs and QTc elongation risk.

Since several drugs cause QTc elongation, with this case series we want to analyze if QTc lengthening occurs on a total of 74 patients admitted to the Addiction Medicine Unit in Verona, Italy for daily use of high doses of Zolpidem.

## Patients and methods

The study was conducted in the Addiction Medicine Unit of the G.B. Rossi University Hospital in Verona. The data were collected from hospitalizations carried out between January 2015 and February 2020 and refer to a total of 74 patients, of which 38 were males and 36 were females, that were being treated for detoxification from high doses of Zolpidem with “Verona Detox Approach With Flumazenil” (30, 31).

The criterion of dependence on high doses of ZDs was defined on the criteria established by the DSM IV-TR, which provide for the presence of continuous use for a period greater than 6 months, a daily intake of Zolpidem greater than at least five times the maximum recommended daily dose and problematic use of ZDs, such as mixing various molecules, increasing dosages, using for pleasure, obtaining them through illegal means or deriving negative social consequences. The dosage assumed was obtained from a drug use history assessment performed by the staff doctors.

The variables examined were demographic ones, that considered age and sex, and clinical ones, that considered the following: type of BDZ used; DDDE (mg); heart rate (HR; bpm); QY (ms); QTc (ms); Na+; K +; Cl-; additional therapy other than the BDZ.

Upon admission, blood samples were taken to assess electrolyte concentration and an electrocardiogram was performed in order to obtain HR and the QT interval. QT was corrected with HR using Bazett’s formula : QTc = QT/Vrr.

## Results

A total of 74 patients including 38 males and 36 females (Table 1) that were hospitalized, between January 2015 and February 2020, to the Addiction Medicine Unit of the G.B. Rossi University Hospital in Verona, Italy.

**TABLE 1:** Gender.

Gender	Frequency	Percentage
Male	38	51.35
Female	36	48.65
Total	74	100

The average age of these patients was 44 years (SD 11,77) with a minimum of 23 and a maximum of 74 years; 38 were workers, 25 were unemployed and 11 were students (Table 2).

**TABLE 2:** Status.

Status	Frequency	Percentage
Worker	38	51.35
Unemployed	25	33.78
Other (student, retired)	11	14.86
Total	74	100

The mean daily dose of Zolpidem was 402.36 mg/day (SD ± 368.88) (Table 3), with a minimum of 20 mg/day and a maximum of 2,250 mg/day, while the mean DDD is 50.21 mg/day.

**TABLE 3:** Drug dosage.

	N	Minimum	Maximum	Mean	SD
Dosage mg (mean)	74	20	2,250	402.36	368.88
DDD	74	2	300	50.21	58.61

Assuming that the threshold value of QTc is 470 ms in males and 480 in females, we recorded a single case exceeding these threshold values (32). The patient was a male presenting a QTc of 479 ms and taking 50 mg/day of Zolpidem (25 mg of Diazepam equivalents daily dose); he also did not have concomitant therapies that could have caused QTc lengthening, no electrolyte alterations (Na 140 mmol/L, K + 3.75 mmol/L, Cl- 105 mmol / L), no contemporary or previous intake of barbiturates, heroin, cocaine, THC, alcohol, NMDA or nicotine.

It should also be noted that the QTc value of one of the 74 patients was not recorded.

## Discussion

Zolpidem is a non-benzodiazepine receptor modulator used in short-term treatment of insomnia aimed at patients with difficulty starting sleep; it improves measures of sleep latency, sleep duration, and reduces the number of awakenings in patients with transient insomnia. It also improves sleep quality in patients with chronic insomnia and can act as a minor muscle relaxant. (33–35).

The main thing to consider when starting patients on Zolpidem is the possibility of addiction and the development of withdrawal symptoms following drug dismission if the drug has been taken for a long time. An interprofessional team approach comprising healthcare professionals such as a pharmacist, a therapist, a nurse, and a clinician is critical; patients should also be educated on possible withdrawal effects resulting from the medication. Because Zolpidem has the potential to cause dependence, it should not be prescribed for long periods of time.

Moreover, long term use of Z-drugs causes cognitive impairments: several case control studies report that benzodiazepine or Z-drug use approximately doubles the risk of being involved in a motor vehicle accident (7, 36). Other effects may include dependence (37), and next-day cognitive, memory, psychomotor and balance impairments (38).

There is a mistaken belief that Z-drugs have advantages both in terms of efficacy and safety compared to BDZs, and, for this reason, many countries have seen a lower prescription of BDZs in favor of an increase in prescriptions of Z-drugs, especially Zolpidem. However, even this class of drugs can have important side effects, such as reduced functionality of cognitive abilities and worsening of psychomotor performances (1–4, 6, 8, 9).

Inadequate prescribing (non-therapeutic high-dosage administration, long duration of the treatment, association with different drugs, absence of monitoring) and poor overall assessment of the risks associated with their prescribing carries many potential risks to patients, including the risk of developing addiction.

Several drugs have been proven or suspected to cause QT prolongation; many of these medications are frequently used in the intensive care unit (ICU), such as different types of anesthetics, sedatives, antibiotics, antimycotics, antidepressants and antipsychotics. The mechanism behind drug-induced QT prolongation is primarily the blockade of potassium ion channels (Ikr) in myocytes, causing prolonged cardiac repolarization. A secondary mechanism is the blockade of hepatic drug degradation because of inhibition of the cytochrome P450 enzyme CYP3A4. Awareness on the many medications known to cause drug-induced LQTS is imperative, especially when the drugs are combined in the same patient (39, 40).

In literature there is no study that correlates the use of high doses of Z-drugs, in particular Zolpidem, with the analysis of QTc on the ECG. With this study we analyzed whether there was a correlation between the intake of high doses of Zolpidem and QTc prolongation.

The data collected showed that 1.35% of Zolpidem abusers developed QTc elongation, which corresponds to only one subject of the 74 total (with the exception of the single patients for whom we didn’t record QTc data). We could deduce that Zolpidem does not represent a significant cause of this side effect, but this is the only study in literature that treats this problem with very selective criteria. Regarding the population that was taken into consideration, we can affirm that QTc lengthening can be explained by the simultaneous intake of other drugs that present this problem among their side effects (41, 42).

With the information we have, we are unable to formulate a hypothesis that explains this phenomenon. Perhaps other studies focusing on gender differences of patients taking high doses of BDZs or Z-drugs might be more insightful.

### Limitation

This study presents several limitations: it’s a retrospective study, Zolpidem dosage was reported by subjects, no follow-up was included and the sample size is small.

## Conclusion

The present study shows a low risk of QTc elongation caused by high daily doses of Zolpidem; however, it must be considered that this is the only study currently present in literature and it could be useful to expand the data by analyzing a larger sample. Z-drugs and Zolpidem, proved to be safe drugs considering the risk of QTc lengthening, however the problem of abuse and dependence of this drug represents a growing phenomenon which is often underestimated by healthcare professionals.

## Data availability statement

The raw data supporting the conclusions of this article will be made available by the authors, without undue reservation.

## Ethics statement

The studies involving human participants were reviewed and approved by the ethics committee of the Verona University Hospital (approval code 683CESC). The patients/participants provided their written informed consent to participate in this study.

## Author contributions

SC, LZ, RC, and FL were responsible for the study concept and design. SC, FF, and LZ drafted the manuscript. LZ was responsible for the study methodology. All authors critically reviewed the content and approved the final version of the manuscript for publication.

## References

[1] Hoffmann F. Perceptions of German GPs on benefits and risks of benzodiazepines and Z-drugs. *Swiss Med Weekly.* (2013) 143:w13745. 10.4414/smw.2013.13745 23348819

[2] Islam MM Conigrave KM Day CA Nguyen Y Haber PS. Twenty-year trends in benzodiazepine dispensing in the Australian population. *Intern Med J.* (2014) 44:57–64. 10.1111/imj.12315 24450521

[3] Paulose-Ram R Safran MA Jonas BS Gu Q Orwig D. Trends in psychotropic medication use among U.S. adults. *Pharmacoepidemiol Drug Saf.* (2007) 16:560–70. 10.1002/pds.1367 17286304

[4] Siriwardena AN Qureshi Z Gibson S Collier S Latham M. GPs’ attitudes to benzodiazepine and “Z-drug” prescribing: a barrier to implementation of evidence and guidance on hypnotics. *Br J Gen Pract.* (2006) 56:964–7. 17132386 PMC1934058

[5] Drover DR. Comparative pharmacokinetics and pharmacodynamics of short-acting hypnosedatives: zaleplon, zolpidem and zopiclone. *Clin Pharmacokinet.* (2004) 43:227–38. 10.2165/00003088-200443040-00002 15005637

[6] Becker PM Somiah M. Non-Benzodiazepine receptor agonists for Insomnia. *Sleep Med Clin.* (2015) 10:57–76. 26055674 10.1016/j.jsmc.2014.11.002

[7] Gunja N. In the Zzz zone: the effects of Z-drugs on human performance and driving. *J Med Toxicol.* (2013) 9:163–71. 10.1007/s13181-013-0294-y 23456542 PMC3657033

[8] Stranks EK Crowe SF. The acute cognitive effects of zopiclone, zolpidem, zaleplon, and eszopiclone: a systematic review and meta-analysis. *J Clin Exp Neuropsychol.* (2014) 36:691–700. 10.1080/13803395.2014.928268 24931450

[9] Zammit G. Comparative tolerability of newer agents for Insomnia. *Drug Safety.* (2009) 32:735–48. 10.2165/11312920-000000000-00000 19670914

[10] Hajak G Müller WE Wittchen HU Pittrow D Kirch W. Abuse and dependence potential for the non-benzodiazepine hypnotics zolpidem and zopiclone: a review of case reports and epidemiological data. *Addiction.* (2003) 98:1371–8. 10.1046/j.1360-0443.2003.00491.x 14519173

[11] Liappas IA Malitas PN Dimopoulos NP Gitsa OE Liappas AI Nikolaou CK Zolpidem dependence case series: possible neurobiological mechanisms and clinical management. *J Psychopharmacol.* (2003) 17:131–5. 10.1177/0269881103017001723 12680751

[12] Marsden J White M Annand F Burkinshaw P Carville S Eastwood B Medicines associated with dependence or withdrawal: a mixed-methods public health review and national database study in England. *Lancet Psychiatry.* (2019) 6:935–50. 10.1016/S2215-0366(19)30331-1 31588045 PMC7029276

[13] Rush CR. Behavioral pharmacology of zolpidem relative to benzodiazepines: a review. *Pharmacol Biochem Behav.* (1998) 61:253–69. 10.1016/S0091-3057(98)00102-6 9768560

[14] Schifano F Chiappini S Corkery JM Guirguis A. An insight into Z-Drug abuse and dependence: an examination of reports to the european medicines agency database of suspected adverse drug reactions. *Int J Neuropsychopharmacol.* (2019) 22:270–7. 10.1093/ijnp/pyz007 30722037 PMC6441128

[15] Chattopadhyay AC Shukla L Kandasamy A Benegal V. High-dose zolpidem dependence - Psychostimulant effects? A case report and literature review. *Ind Psychiatry J.* (2016) 25:222–4. 10.4103/ipj.ipj_80_14 28659704 PMC5479098

[16] Sabe M Kashef H Gironi C Sentissi O. Zolpidem stimulant effect: induced mania case report and systematic review of cases. *Prog Neuropsychopharmacol Biol Psychiatry.* (2019) 94:109643. 10.1016/j.pnpbp.2019.109643 31071363

[17] Victorri-Vigneau C Dailly E Veyrac G Jolliet P. Evidence of zolpidem abuse and dependence: results of the French Centre for Evaluation and Information on Pharmacodependence (CEIP) network survey. *Br J Clin Pharmacol.* (2007) 64:198–209. 10.1111/j.1365-2125.2007.02861.x 17324242 PMC2000636

[18] Federico A Mantovani E Casari R Bertoldi A Lugoboni F Tamburin S. Adult attention-deficit/hyperactivity disorder symptoms, cognitive dysfunction and quality of life in high-dose use of benzodiazepine and Z-drug. *J Neural Trans.* (2021) 128:1109–19. 10.1007/s00702-020-02285-w 33331955 PMC8295124

[19] Lugoboni F Bertoldi A Casari R Mantovani E Morbioli L Tamburin S. Adult attention-deficit/hyperactivity disorder and quality of life in high-dose benzodiazepine and related Z-Drug users. *Eur Addict Res.* (2020) 26:274–82. 10.1159/000507852 32570244

[20] Bazett HC. An analysis of the time-relations of electrocardiograms. *Heart.* (1920) 7:353–70.

[21] Jordan MP. *ECG: Basic Principles of ECG Analysis.* Waltham, MA: UpToDate (2019).

[22] Khan IA. Long QT syndrome: diagnosis and management. *Am Heart J.* (2002) 143:7–14. 10.1067/mhj.2002.120295 11773906

[23] Passman R Kadish A. Polymorphic ventricular tachycardia, long Q-T syndrome, and torsades de pointes. *Med Clin North Am.* (2001) 85:321–41. 10.1016/s0025-7125(05)70318-7 11233951

[24] Suessbrich H Schönherr R Heinemann SH Attali B Lang F Busch AE. The inhibitory effect of the antipsychotic drug haloperidol on HERG potassium channels expressed in Xenopus oocytes. *Br J Pharmacol.* (1997) 120:968–74. 10.1038/sj.bjp.0700989 9138706 PMC1564549

[25] Yang T Roden DM. Extracellular potassium modulation of drug block of IKr. implications for torsade de pointes and reverse use-dependence. *Circulation.* (1996) 93:407–11. 10.1161/01.cir.93.3.407 8565156

[26] Drici MD Clément N. Is gender a risk factor for adverse drug reactions? The example of drug-induced long QT syndrome. *Drug Saf.* (2001) 24:575–85. 10.2165/00002018-200124080-00002 11480490

[27] Makkar RR Fromm BS Steinman RT Meissner MD Lehmann MH. Female gender as a risk factor for torsades de pointes associated with cardiovascular drugs. *JAMA.* (1993) 270:2590–7. 10.1001/jama.270.21.2590 8230644

[28] Zeltser D Justo D Halkin A Prokhorov V Heller K Viskin S. Torsade de pointes due to noncardiac drugs: most patients have easily identifiable risk factors. *Medicine.* (2003) 82:282–90. 10.1097/01.md.0000085057.63483.9b 12861106

[29] Ozeki Y Fujii K Kurimoto N Yamada N Okawa M Aoki T QTc prolongation and antipsychotic medications in a sample of 1017 patients with Schizophrenia. *Prog Neuropsychopharmacol Biol Psychiatry.* (2010) 34:401–5. 10.1016/j.pnpbp.2010.01.008 20079791

[30] Casari R Metastasio A Zamboni L Biasioli M Campagnari S Lugoboni F. Addiction of high dose of benzodiazepine: verona detox approach with flumazenil. *Front Psychiatry.* (2022) 13:857376. 10.3389/fpsyt.2022.857376 35432044 PMC9008883

[31] Federico A Lugoboni F Mantovani E Martini A Morbioli L Casari R Detoxification improves multidomain cognitive dysfunction in high-dose benzodiazepine abusers. *Front Neurosci.* (2020) 14:747. 10.3389/fnins.2020.00747 32848544 PMC7396668

[32] Priori SG Blomström-Lundqvist C Mazzanti A Blom N Borggrefe M Camm J 2015 ESC Guidelines for the management of patients with ventricular arrhythmias and the prevention of sudden cardiac death: the task force for the management of patients with ventricular arrhythmias and the prevention of sudden cardiac death of the European Society of Cardiology (ESC). Endorsed by: Association for European Paediatric and Congenital Cardiology (AEPC). *Eur Heart J.* (2015) 36:2793–867. 26320108 10.1093/eurheartj/ehv316

[33] Bjurström MF Irwin MR. Perioperative pharmacological sleep-promotion and pain control: a systematic review. *Pain Pract.* (2019) 19:552–69. 10.1111/papr.12776 30762974

[34] Kim HM Gerlach LB Van T Yosef M Conroy DA Zivin K. Predictors of long-term and high-dose use of zolpidem in veterans. *J Clin Psychiatry.* (2019) 80:18m12149. 10.4088/JCP.18m12149 30840786

[35] Sharma MK Kainth S Kumar S Bhardwaj A Agarwal HK Maiwall R Effects of zolpidem on sleep parameters in patients with cirrhosis and sleep disturbances: a randomized, placebo-controlled trial. *Clin Mol Hepatol.* (2019) 25:199–209. 10.3350/cmh.2018.0084 30856689 PMC6589852

[36] Thomas RE. Benzodiazepine use and motor vehicle accidents. Systematic review of reported association. *Can Fam Phys.* (1998) 44:799–808. PMC2277821 9585853

[37] Lugoboni F Mirijello A Faccini M Casari R Cossari A Musi G Quality of life in a cohort of high-dose benzodiazepine dependent patients. *Drug Alcohol Depend.* (2014) 142:105–9. 10.1016/j.drugalcdep.2014.06.020 25001277

[38] Mets MA de Vries JM de Senerpont Domis LM Volkerts E Olivier B Verster JC. Next-day effects of ramelteon (8 mg), zopiclone (7.5 mg), and placebo on highway driving performance, memory functioning, psychomotor performance, and mood in healthy adult subjects. *Sleep.* (2011) 34:1327–34. 10.5665/SLEEP.1272 21966064 PMC3173578

[39] Beitland S Platou ES Sunde K. Drug-induced long QT syndrome and fatal arrhythmias in the intensive care unit. *Acta Anaesthesiol Scand.* (2014) 58:266–72. 10.1111/aas.12257 24397608

[40] Kallergis EM Goudis CA Simantirakis EN Kochiadakis GE Vardas PE. Mechanisms, risk factors, and management of acquired long QT syndrome: a comprehensive review. *ScientificWorldJournal.* (2012) 2012:212178. 22593664 10.1100/2012/212178 PMC3347892

[41] Katchman AN Koerner J Tosaka T Woosley RL Ebert SN. Comparative evaluation of HERG currents and QT intervals following challenge with suspected torsadogenic and nontorsadogenic drugs. *J Pharmacol Exp Therap.* (2006) 316:1098–106. 10.1124/jpet.105.093393 16278312

[42] Ray WA Murray KT Meredith S Narasimhulu SS Hall K Stein CM. Oral erythromycin and the risk of sudden death from cardiac causes. *N Engl J Med.* (2004) 351:1089–96. 10.1056/NEJMoa040582 15356306

